# Non-Destructive Assessment of Watermelon Comprehensive Quality Based on Acoustic and Vibration Signals

**DOI:** 10.3390/s26134000

**Published:** 2026-06-24

**Authors:** Wenyu Li, Qihan Wang, Xi Lin, Shuaiqi Guo, Meng Ma

**Affiliations:** 1 School of Civil Engineering, Beijing Jiaotong University, Beijing 100044, China; 23231173@bjtu.edu.cn (W.L.); 23121013@bjtu.edu.cn (S.G.); 2 School of Electronic and Information Engineering, Beijing Jiaotong University, Beijing 100044, China; 23211194@bjtu.edu.cn; 3 School of Traffic and Transportation, Beijing Jiaotong University, Beijing 100044, China; 23251108@bjtu.edu.cn

**Keywords:** watermelon, comprehensive quality, non-destructive testing, acoustic and vibration signal, extra-trees, factor analysis of mixed data

## Abstract

The internal quality of watermelons has garnered extensive attention. Conventional destructive quality detection for watermelons causes fruit loss, while existing acoustic techniques often rely on a single evaluation index. To address these issues, this study proposes a non-destructive method for comprehensive watermelon quality detection using acoustic and vibration signals. Signals from two watermelon varieties were collected under impact excitation to extract six time-domain and frequency-domain features. Factor Analysis of Mixed Data (FAMD) was employed to integrate ripeness, Soluble Solids Content (SSC), firmness, and sensory scores into a Comprehensive Quality Index (CQI), categorizing samples into High-Quality, Medium-Quality, and Low-Quality groups. Following physically constrained data augmentation to mitigate small sample size and class imbalance, an Extremely Randomized Trees (Extra-Trees) model was constructed. Results demonstrate that the Extra-Trees model achieved an overall testing accuracy of 0.92, with recall rates of 0.93 and 1.00 for Low-Quality and High-Quality watermelons, respectively. Recognition for Medium-Quality samples was lower due to overlapping physical and acoustic characteristics. Ultimately, this system aligns with actual consumer demands, providing technical support for low-cost, portable, and non-destructive watermelon grading.

## 1. Introduction

Watermelon is a globally cultivated horticultural crop of significant economic importance. As a staple fruit for summer heat relief, its internal quality directly dictates its market value and consumers’ sensory experience. Driven by rising living standards, both market and consumer expectations for watermelon quality have continuously increased. Growers and distributors must guarantee product quality, while consumers increasingly demand superior taste and texture. Conventional quality assessment predominantly relies on destructive methods—such as sectioning, juicing and other destructive detection approaches—which result in substantial fruit loss and are impractical for application in consumption scenarios. Consequently, the development of rapid, accurate non-destructive testing (NDT) technologies for watermelon quality evaluation holds profound theoretical value and practical significance.

Among various NDT modalities, including near-infrared spectroscopy (NIRS), X-ray imaging, and nuclear magnetic resonance (NMR), methods based on acoustic and vibration signals offer distinct advantages in terms of low equipment cost, facility for mobile and intelligent deployment [1,2]. In their review, Jie and Wei [3] noted that acoustic methods are cost-effective, highly sensitive, and adaptable for the non-destructive testing of watermelon internal quality, though they are susceptible to environmental interference. Ghosh et al. [4] systematically summarized the application of acoustic and vibration techniques coupled with artificial neural networks (ANN) and machine learning algorithms in fruit quality evaluation, emphasizing their potential in evaluating internal parameters of fruit quality such as texture, firmness, and crispness. The review by Caceres-Hernandez et al. [5] focused on automated watermelon identification systems based on imaging, acoustic, and spectroscopic technologies, providing theoretical support for multi-technology integration.

Research on acoustic-based non-destructive testing for watermelon quality dates back to the 1980s. Yamamoto et al. [6] investigated the use of impact excitation to extract the fundamental frequencies of apples and watermelons. Their findings revealed that while the acoustic characteristics of apples correlated significantly with their elastic modulus, ultimate strength, and firmness, such correlations were comparatively low for watermelons. However, the mass-compensated acoustic index still proved practically useful. Since then, researchers globally have conducted systematic studies focusing on the optimization of detection points, sensor selection, and data analysis methodologies.

To optimize the selection of detection points, Diezma [7] utilized vibration characteristics, modal analysis, and finite element analysis (FEA) to determine that the ideal excitation-reception configuration lies at diametrically opposed positions on the equator. Through comparative experiments, Wei et al. [8] verified the superiority of predictive models based on equatorial point configurations. Abbaszadeh et al. [9] found that under the first resonant mode, the maximum deformation amplitude and the most intense vibration occur at the midpoints of the upper and lower sides of the watermelon. Considering varietal differences, Wang et al. [10] found that for their specific cultivar, acoustic characteristics correlated more strongly with internal sugar content when using the stem end for excitation and the blossom end for reception. Furthermore, Alipasandi et al. [11] systematically investigated the impact of the excitation location, as well as the material and diameter of the striking ball, on classification performance. They demonstrated that striking the stem end with a small metal ball achieved an optimal classification accuracy of 77.3%, providing an empirical basis for designing portable devices.

Regarding sensor selection, Abbaszadeh et al. [12] and Cui et al. [13] applied continuous vibrations at specific frequencies using shakers and loudspeakers, combined with Laser Doppler Vibrometer (LDV) scanning, to obtain high-precision frequency response functions. However, due to expensive equipment and slow detection speeds, this method is more suitable for detailed laboratory analysis. Mao et al. [14] evaluated how different materials for the impact ball and the supporting fruit tray affected testing performance. They found that combining a high-elastic-modulus stainless steel ball with a plastic tray minimized the standard deviation of the measured resonance frequencies, thereby maximizing signal stability. In a comprehensive review, Yu et al. [2] noted that microphones were adopted in nearly half of the relevant studies due to their non-contact nature and deployment flexibility. Additionally, Zhang et al. [15] highlighted that accelerometers, as the predominant contact sensors, can be directly affixed to the watermelon rind to measure vibration acceleration. This direct contact provides distinct advantages, including robust signal strength and strong immunity to airborne noise interference.

With the rapid development of information technology, analytical methods for the acoustic and vibration signals of watermelons have undergone continuous upgrades. Early studies primarily focused on time-domain waveforms and fundamental frequency characteristics. He et al. [16] observed that the acoustic waveform generated by mechanical impact changes systematically with watermelon maturity, while Wang et al. [17] verified the correlation between the fundamental frequency and soluble solids content (SSC). Subsequently, researchers gradually adopted more sophisticated signal processing and machine learning techniques. Taniwaki et al. [18] employed a non-destructive acoustic and vibration method to monitor changes in the elasticity index of melons. By determining the maturation rate and identifying the optimal ripeness window, they demonstrated the effectiveness of acoustic techniques in the dynamic monitoring of melon maturity. To simulate human auditory perception, Baki et al. [19] utilized Mel-Frequency Cepstral Coefficients (MFCC) for feature extraction. By combining these features with a Multilayer Perceptron (MLP) neural network, they classified watermelons into mature and immature categories with an accuracy of 77.25%. Gao et al. [20] proposed the Band Magnitude Vector (BMV) feature and employed a Probabilistic Neural Network (PNN) to detect the maturity of two watermelon cultivars, achieving accuracies of 86.96% and 91.67%, respectively. Wei et al. [8] selected the acoustic transmittance at six characteristic frequencies to establish a multiple linear regression model for SSC prediction, attaining a calibration correlation coefficient of 0.81. Ikeda et al. [21] developed a watermelon flesh firmness evaluation method based on surface elastic waves. By measuring the surface wave propagation velocity within the 800–2400 Hz frequency range and calculating the shear elastic modulus, they found that the surface wave velocity decreased by approximately 10% after 10 days of storage. This finding was highly correlated with sensory firmness evaluations, providing direct experimental validation for the physical significance of time-domain acoustic and vibration features (such as vibration decay time).

Chen et al. [22] proposed a watermelon maturity detection method based on wavelet multi-resolution decomposition and statistical hypothesis testing. This approach eliminates the need for complex feature extraction and classification modeling, achieving a detection accuracy of 91.76%. However, because it only distinguishes between mature and immature states, it is primarily applicable to rough sorting. Li et al. [23] proposed a signal processing methodology for transient percussion signals that integrated Non-negative Matrix Factorization (NMF) filtering with root-mean-square normalization. By employing the Hilbert–Huang Transform (HHT) to analyze frequency characteristics, they combined NMF with a Support Vector Machine (SVM) and achieved a classification accuracy of 92.22%, significantly surpassing the performance of conventional approaches. Finally, Nayana et al. [24] systematically compared the performance of various machine learning algorithms in predicting watermelon maturity, revealing that SVM and Light Gradient Boosting Machine (LightGBM) exhibited superior consistency and robustness, providing a reference basis for algorithm selection.

In recent years, machine learning and deep learning technologies have been extensively adopted. Chawgien et al. [25] demonstrated that gradient boosting tree models can achieve a classification accuracy of up to 92% for watermelon sugar content. Choe et al. [26] assessed watermelon maturity by measuring acoustic wave propagation velocity using piezoelectric transducers, and when combined with artificial neural network (ANN) modeling, the coefficient of determination (R^2^) for sound velocity reached 98.70%. Zuo et al. [27] developed a full-variable classification model based on decision trees, Naïve Bayes, support vector machines (SVM), and K-nearest neighbors (KNN) to relate frequency-domain signals to three-tier watermelon quality grades. Their model achieved a grading accuracy of 95.56% on the validation set and realized a full recall of Grade II products, which aligns more closely with practical consumer market demands. Furthermore, Ke [28] directly input frequency-domain signals into a customized convolutional neural network (CNN), allowing the model to autonomously learn features. This approach yielded a maturity classification accuracy of 99.69%, outperforming KNN and SVM algorithms that rely on manual feature engineering.

Koç and Akbalik [29] reported that a Random Forest Classifier (RFC) achieved an accuracy of 98.2% for classifying mature watermelons, while a K-Nearest Neighbor Classifier (KNC) distinguished overripe and unripe samples with accuracies of 96.3% and 96.2%, respectively. Breaking away from traditional binary classification (mature vs. unripe), Zhang et al. [30] categorized watermelons into four classes: mature, unripe, hollow-heart, and juicy. Using the Rep-MBF model, they accurately predicted 44 out of 48 samples, achieving an overall detection success rate of 91.67%.

In terms of developing practical detection systems, both academia and industry have steadily advanced the real-world application of acoustic non-destructive testing for watermelons. Pamungkas and Bintoro [31] developed a simple, portable maturity detector based on the fundamental frequency and amplitude of acoustic signals. Alipasandi et al. [11] designed a low-cost portable sensor suitable for rapid field sorting of watermelons. Zeng [32] and Ke [28] integrated detection systems with mobile devices by encapsulating machine learning models into smartphone applications. By simply recording the sound of a tap with a smartphone, users can rapidly assess watermelon quality, significantly lowering the technical threshold for practical use. Li et al. [33] further investigated the factors influencing maturity classification using smartphone-acquired acoustic signals. Their findings revealed that cultivar had the most significant impact, followed by the acquisition site and striking intensity. Specifically, signals collected at the equatorial region yielded the best classification performance, providing theoretical guidance for mobile application development. Additionally, Zou et al. [34] explored the application of photoacoustic (PA) detection technology in watermelon maturity testing, assessing quality by measuring rind thickness and flesh redness, thereby offering a novel technical complement to conventional acoustic detection.

Despite the substantial progress achieved in acoustic-based non-destructive testing of watermelon quality, several limitations still persist. First, most high-precision detection models rely on pre-screened samples from a single cultivar, origin, and batch. Consequently, their generalizability across diverse varieties and production regions remains unverified. Second, a watermelon’s commercial value and consumer purchase intent are driven by multidimensional quality attributes. Beyond sugar content, attributes such as flesh texture, juiciness, rind thickness, and the presence of internal defects (e.g., hollow-heart) collectively constitute the overall evaluation of a superior watermelon. However, current research predominantly employs SSC or maturity as isolated evaluation metrics, lacking a quantitative characterization of comprehensive quality. Furthermore, most studies are limited to binary classification or rough three-tier grading schemes and fail to capture nuanced consumer preferences for textures, such as mealiness versus crispness. Finally, because acoustic and vibration signals are mechanical waves, their propagation characteristics are primarily governed by the material’s density and elastic modulus. However, the accumulation of chemical components, such as SSC, exerts a negligible impact on the elastic modulus. This indicates that acoustic methods may have inherent limitations in distinguishing samples with marginal quality differences—an issue rarely explored in depth in existing literature.

To address these limitations, this study used ‘Beijing Tianwang’ and ‘Shanxi Xiabao’ watermelons as experimental subjects to develop a non-destructive testing methodology based on acoustic and vibration signals and Extremely Randomized Trees (Extra-Trees), specifically designed for small-sample scenarios. The main research contents are as follows:

(1) Constructing a comprehensive watermelon quality evaluation system. Ripeness scores, soluble solids content (SSC), firmness, and sensory evaluation scores were simultaneously acquired. Factor Analysis of Mixed Data (FAMD) was used to reduce the dimensionality of these multidimensional indicators and to derive a Comprehensive Quality Index (CQI), which served as the target label for subsequent classification models.

(2) Extracting acoustic and vibration features and analyzing quality response mechanisms. An experimental platform was constructed to capture the acoustic and vibration signals generated by impact excitation. Features such as time-domain energy, the fundamental frequency and Mel-Frequency Cepstral Coefficients (MFCC) were extracted. Guided by principles of mechanics, this study systematically analyzed the acoustic and physical mechanisms underlying feature variations induced by internal quality deterioration.

(3) Developing a small-sample classification model based on Extra-Trees. To reduce the overfitting risk associated with the small sample size, a physically constrained data augmentation method was applied to expand the dataset. An Extra-Trees multi-class classification model was then constructed and compared with a Support Vector Classifier (SVC), Random Forest (RF), and Light Gradient Boosting Machine (LightGBM) to verify the robustness of the extracted features.

(4) Analyzing the physical limitations of acoustic detection and evaluating its commercial values. Based on the model’s decision ambiguity when distinguishing between adjacent quality grades (i.e., Medium-Quality vs. High-Quality) and considering the gradual transition of the fruit’s physicochemical properties, this study discusses the limitations of acoustic waves—as mechanical waves—in the fine-grained non-destructive evaluation of agricultural products. The potential commercial value of this methodology is also assessed.

## 2. Materials and Methods

**Figure 1:**
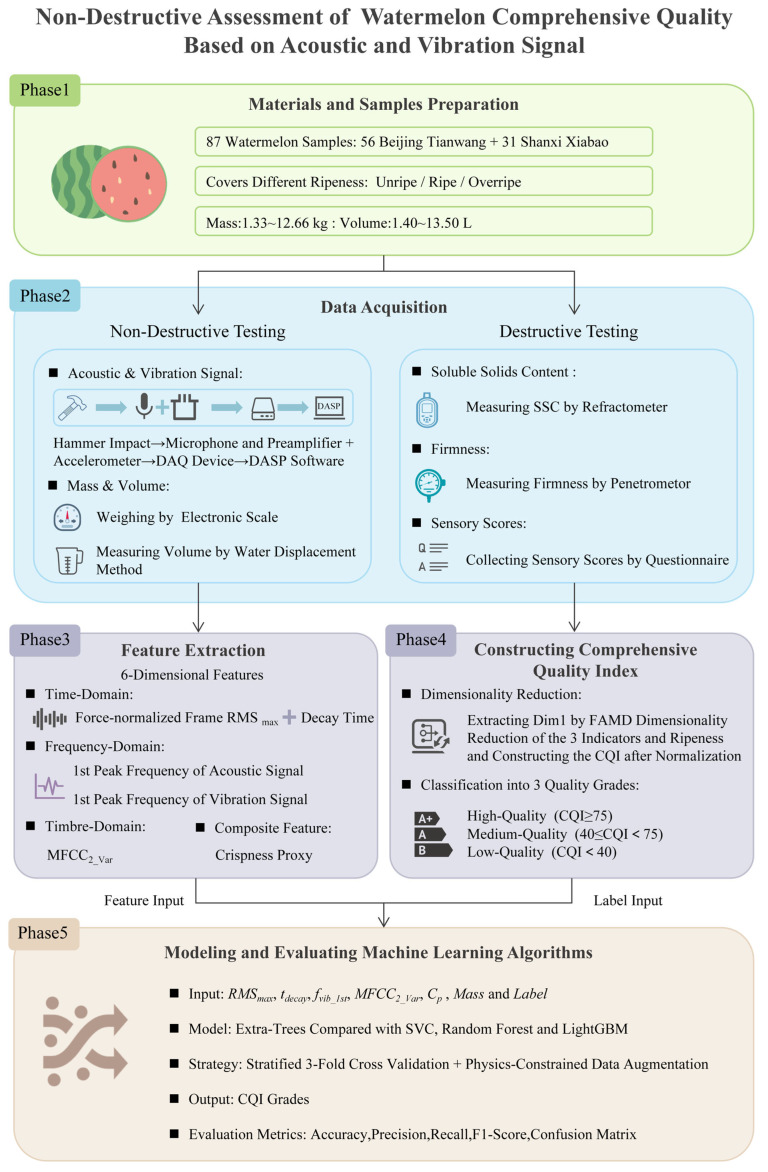
Overall technical flowchart of non-destructive comprehensive quality assessment of watermelon.

The overall technical workflow of this study is illustrated in Figure 1, comprising five phases: materials and samples preparation, data acquisition (non-destructive testing and destructive validation conducted in parallel), feature extraction, construction of the Comprehensive Quality Index (CQI), and machine learning modeling and evaluation. Acoustic and vibration features acquired via non-destructive testing served as model inputs, while the CQI, derived from destructive validation and FAMD, acted as the classification label. Ultimately, algorithms such as Extra-Trees were employed to achieve the non-destructive grading of watermelon quality.

### 2.1. Experimental Materials

Two commercially prevalent cultivars, “Beijing Tianwang” and “Shanxi Xiabao”, were selected as research subjects. The former is a long-elliptical, thin-rind, high-sugar type, whereas the latter is a short-elliptical, thick-rind type with good storage and transportation tolerance. These two cultivars exhibit substantial divergence in fruit shape index, rind thickness, flesh texture, and ripening characteristics, encompassing the principal range of physical properties in watermelons and ensuring cultivar representativeness.

A total of 87 watermelon samples (56 “Beijing Tianwang” and 31 “Shanxi Xiabao”) were collected in multiple batches across different seasons. They were sourced from local supermarkets, suburban agricultural fields, and e-commerce platforms to account for individual variations arising from origin, growing conditions, and harvest maturity. Based on ripeness classification derived from subsequent destructive validation, the sample set included 3 unripe, 55 ripe, and 29 overripe watermelons. With respect to procurement source, 54 samples were obtained from supermarkets, 4 via direct field collection, and 29 through online e-commerce channels.

The sample mass ranged from 1.33 to 12.66 kg (mean ± SD: 5.52 ± 2.76 kg), and the volume ranged from 1.40 to 13.50 L (5.87 ± 2.85 L). These two parameters exhibited a strong positive correlation, forming a continuous gradient that effectively captures the physical variations driven by cultivar, size, and ripeness. Importantly, the ripeness categories (unripe, ripe, and overripe) were visually assessed based on flesh color after cutting. This visual classification should be distinguished from the comprehensive quality grades (High-Quality, Medium-Quality, and Low-Quality) derived from the CQI detailed in Section 3.1.

### 2.2. Experimental Apparatus

The experimental procedure consisted of two main phases: non-destructive testing and destructive validation. In the non-destructive phase, acoustic-vibration signals, mass, and volume were recorded prior to cutting, serving as feature inputs for the prediction model. Subsequently, the destructive validation phase measured post-cutting SSC and firmness, which along with subjective sensory scores established the ground truth for quality assessment. Together, these paired datasets provided a complete foundation for mapping acoustic-vibration features to overall watermelon quality.

**Figure 2:**
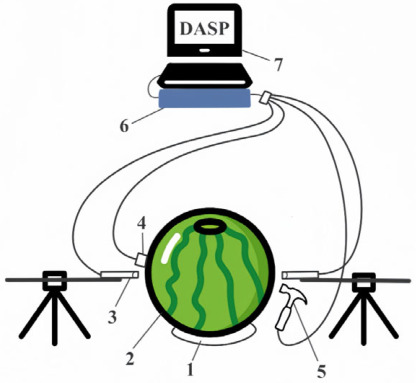
Schematic diagram of the acoustic and vibration signal acquisition system. 1—sample support frame; 2—Sample; 3—measurement microphone and preamplifier; 4—Accelerometer; 5—Handheld impact hammer; 6—Data acquisition unit; 7—Computer.

The acoustic and vibration signal acquisition system is shown in Figure 2, consisting of a sample support frame, a handheld impact hammer, two measurement microphones and preamplifiers, an accelerometer, a data acquisition unit, and a computer equipped with DASP signal analysis software.

The sample support frame consists of a standard plastic ring commonly used for displaying watermelons in supermarkets. This setup simulates authentic commercial conditions while minimizing interference with measurement accuracy [14]. Furthermore, the handheld impact hammer features an aluminum head to generate a clear and pronounced excitation signal. It also incorporates a built-in force transducer to monitor the impact force in real time, ensuring consistent excitation.

Acoustic signals were acquired using two MPA 231 1/2-inch pre-polarized measurement microphones paired with MA231 microphone preamplifiers (BSWA TECH, Beijing, China; sensitivity: 32.0 mV/Pa) positioned on the impact side and the opposite side, respectively. This dual placement enabled a comparative analysis of different reception locations. Simultaneously, vibration signals were recorded using an accelerometer (SNz897; sensitivity: Lance Technologies Inc., Qinhuangdao, China; 500 mV/g; range: ±10 g). To capture vibration transmission across the entire cross-section and obtain richer structural information, the accelerometer was firmly affixed to the side opposite the impact point.

All acquired signals were routed to a computer via a data acquisition unit (INV3062S; China Orient Institute of Noise & Vibration, Beijing, China). Real-time signal display, storage, and preliminary analysis were managed using DASP software (DASP-V10; China Orient Institute of Noise & Vibration, Beijing, China; sampling frequency: 2048 Hz; Anti-Aliasing Filter (AF): 800 Hz).

Regarding physical characteristics, sample mass was measured with a precision electronic scale (Model CFC-HR-S01; Haier, Qingdao, China; accuracy: 0.001 kg), while volume was determined via the water displacement method using a 20 L graduated vessel.

For internal quality assessment, SSC was quantified using a refractometer (Model AK002B, AIOK Instrument Co., Ltd., Shenzhen, China), and firmness was evaluated with a dial penetrometer (Model GY-3, Weidu Electronics Co., Ltd., Wenzhou, China), with results recorded in kg/cm^2^. Finally, subjective sensory evaluation was conducted via a questionnaire, where tasters rated sweetness, firmness, juiciness, and overall satisfaction on a 5-point scale. Detailed survey methodology is provided in Section 2.3.4.

### 2.3. Data Acquisition Process

#### 2.3.1. Acoustic-Vibration Signal Acquisition Method

**Figure 3:**
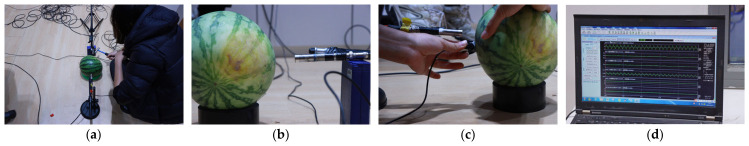
Photographs of the acoustic and vibration signal acquisition process: (**a**) impact striking; (**b**) sound level meter; (**c**) accelerometer; (**d**) DASP software.

Acoustic signals were acquired from watermelons in a quiet, room-temperature indoor environment, as shown in Figure 3. For each sample, two sets of excitation–reception points were established: the first set was located along the equatorial axis (on one side and its diametrically opposite side), and the second set was positioned at the stem and blossom ends.

Before acquisition, each sample was placed steadily on the circular watermelon support frame and adjusted to ensure stability. To minimize operator variability, each measurement point was struck five times with a handheld impact hammer at a uniform velocity and consistent force by a single experimenter. The impact force was monitored in real time by an integrated force sensor on the DASP software, ensuring that the input impact force and wave profiles remained highly uniform and close to each other across all samples. To ensure data stability and mitigate random manual errors, three consistent signals were selected for feature extraction, and their respective feature values were averaged to represent the sample. The remaining two strikes were reserved as backups to account for any potential collection anomalies or sensor failures.

During the excitation process, sound pressure signals were simultaneously captured from both the impact and opposite sides using two sound level meters, alongside vibration signals recorded from the opposite side via an accelerometer. All signals were continuously routed in real time through a data acquisition unit to a computer, where they were sampled, visualized, and stored using DASP software. Finally, the recorded data were exported to Excel format for subsequent processing and feature extraction.

#### 2.3.2. Measurement Methods for Mass and Volume

**Figure 4:**
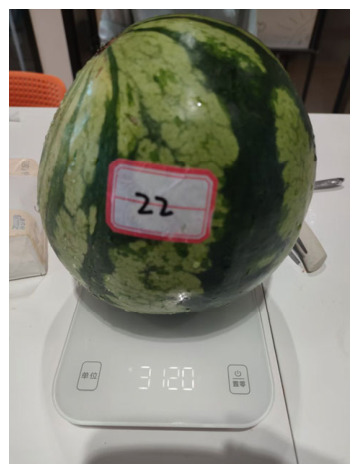
Mass measurement of watermelon using an electronic balance.

Sample mass was determined using a tared electronic balance placed on a level surface (Figure 4). Each watermelon was centered on the platform, and its weight (in kg) was recorded upon stabilization.

**Figure 5:**
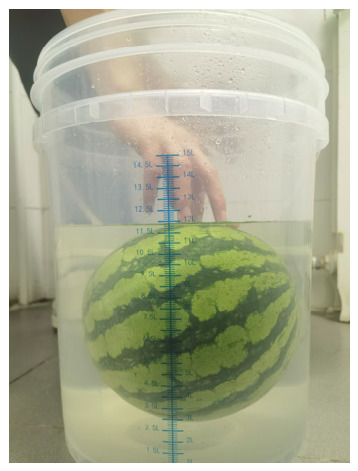
Volume measurement of watermelon by the water displacement method.

Sample volume was determined via water displacement (Figure 5). Initially, a graduated vessel was filled with sufficient water to establish a baseline reading. Each watermelon was then carefully submerged without causing overflow. Upon water level stabilization, a final reading was recorded. The difference between these two values yielded the sample volume (in L). For accuracy, all measurements were performed in triplicate, with the averages serving as the final recorded volumes.

#### 2.3.3. Measurement of Soluble Solids Content, Firmness, and Determination of Ripeness

Following non-destructive testing, each watermelon sample was halved to measure its soluble solids content (SSC) and firmness, and to preliminarily assess its ripeness based on flesh color.

**Figure 6:**
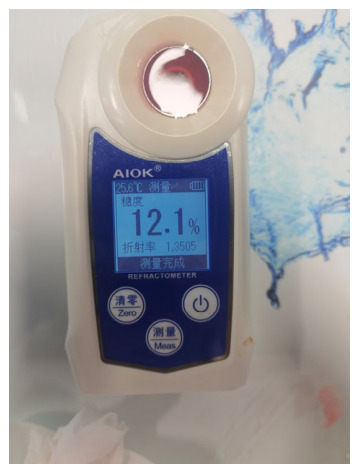
Refractometer for SSC measurement. (Screen display: 25.6 °C, Brix: 12.1%, Refractive index: 1.3505, Measurement complete).

SSC was determined using a refractometer (Figure 6). After calibrating the instrument, the crushed tissue was allowed to settle briefly to isolate crude fibers and suspended pulp solids. A single drop of the pure, clear supernatant juice was carefully sampled using a lab spoon (ensuring no visible fiber residue remained) and placed on the refractometer prism. The SSC value, expressed as the sucrose-equivalent concentration (in Brix), was recorded once the reading stabilized.

**Figure 7:**
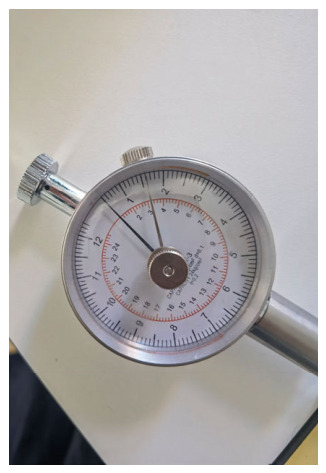
Dial penetrometer for firmness measurement.

Firmness was evaluated using a dial penetrometer (Figure 7). The probe was pressed vertically into the flesh at a constant speed, and the firmness value (in kg/cm^2^) indicated on the dial was recorded when the probe reached the measurement line.

To ensure a comprehensive representation of overall fruit quality, these measurements were taken at three distinct locations for each sample: the central, middle, and near-rind regions of the flesh.

Ripeness was classified into three grades based on flesh coloration after cutting, combined with SSC and firmness measurements: (1) Unripe fruit: characterized by pale, whitish or slightly reddish flesh, incomplete internal development, predominantly white or immature seeds, astringent taste, low SSC, and high firmness. (2) Ripe fruit: characterized by bright red flesh, complete internal development, and crisp, sweet, palatable taste. (3) Overripe fruit: characterized by flesh decay, water-soaking or sponginess, or internal feeding damage by pests, sour taste or off-flavors, abnormally decreased SSC, and significantly reduced firmness.

**Figure 8:**
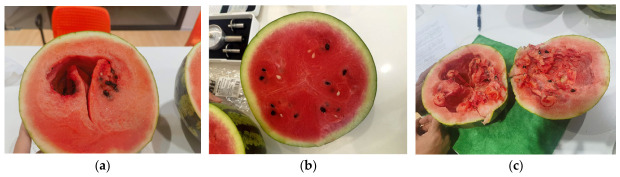
Cross-sectional examples of watermelons at different ripeness grades: (**a**) unripe; (**b**) ripe; (**c**) overripe.

Photographs of representative samples are presented in Figure 8.

To comprehensively evaluate watermelon quality, sensory evaluation surveys were conducted to obtain sensory feedback concurrently with objective indicator measurements. The sensory evaluations and objective indicators collectively constituted the ground truth for the comprehensive quality assessment system.

#### 2.3.4. Sensory Evaluation Methodology

A subjective sensory evaluation questionnaire was designed with four assessment indices: sweetness, firmness, juiciness, and overall satisfaction. Each index was rated on a 5-point Likert scale: 1 denoted “very poor/very dissatisfied,” 2 “poor/dissatisfied,” 3 “neutral,” 4 “good/satisfied,” and 5 “excellent/very satisfied.” The questionnaire recorded both samples and tasters’ identification numbers to facilitate subsequent data matching.

A total of 50 tasters were recruited for the sensory evaluation, comprising 22 males and 28 females, aged 18–50 years.

Edible watermelon samples (unripe and ripe watermelons) were cut open, peeled, and portioned into uniformly sized cubes (approximately 3 cm × 3 cm × 3 cm). These cubes were then placed in standardized food-grade containers, labeled with a questionnaire and sample identification numbers. Overripe and spoiled watermelons were excluded from sensory evaluation due to safety concerns associated with rot and off-odors. Each sample was independently evaluated by at least 12 tasters to mitigate the influence of individual subjective preferences.

Tasters independently completed the questionnaires based on their tasting experiences. Once all questionnaires were collected, the arithmetic means of all ratings for each index were calculated per sample to obtain final sensory evaluation scores (units: points, one decimal place retained). These sensory evaluation scores, together with SSC, firmness, and ripeness measurements, were integrated to construct the subsequent Comprehensive Quality Index.

### 2.4. Feature Extraction

Based on the correlation analysis, acoustic and vibration signals acquired from the opposite side of the equator were selected for preprocessing and feature extraction. This step transformed the raw time-domain signals into specific features that are indicative of watermelon quality.

A total of six features were extracted to serve as input variables for subsequent modeling. The time-domain features comprised the force-normalized maximum frame RMS energy (*NRMS*_max_) and vibration decay time (*t*_decay_). The frequency-domain features included the fundamental frequencies of the acoustic signal from the opposite side (*f*_farsound_1st_) and the vibration signal (*f*_vib_1st_). Since the correlation coefficient between *f*_vib_1st_ and Comprehensive Quality Index (CQI) is slightly lower than that between *f*_farsound_1st_ and CQI, we selected *f*_farsound_1st_ as an independent feature to mitigate multicollinearity, while using *f*_vib_1st_ in conjunction to construct a composite “Crispness Proxy” index. Additionally, the variance of the second Mel-frequency cepstral coefficient (*MFCC*_2_var_) was extracted to represent the acoustic timbre. Due to the sample volume’s low correlation coefficient with CQI, it was removed.

All data processing and feature extraction procedures were implemented using PyCharm 2024.1 (Python 3.12.1) and OriginPro 2024.

#### 2.4.1. Time-Domain Features

The force-normalized maximum frame RMS energy (*NRMS*_max_) and vibration decay time (*t*_decay_) generally reflect the damping characteristics of an object. When watermelon flesh becomes overripe, leading to the degradation of pectin and cell walls, or when hollow hearts and cracks develop, the internal damping increases significantly. This causes rapid energy attenuation during acoustic wave propagation. Consequently, these two features can quantitatively characterize the internal compactness and structural integrity of the watermelons.

The root-mean-square (*RMS*) reflects the energy level of a signal and is defined as the square root of the mean of the squared signal amplitudes:

(1) $$RMS=\sqrt{\frac{1}{N}\sum_{i=1}^{N}{x_{i}}^{2}}$$

where *x_i_* denotes the signal amplitude at the *i*-th sample, and *N* represents the total number of samples.

The maximum frame *RMS* energy characterizes the peak energy level of the acoustic and vibration signal, reflecting the maximum transmission intensity of the impact energy. This feature is extracted using a sliding-window framing strategy. Specifically, the raw vibration signal is segmented using a frame length of 500 samples and a frame shift of 250 samples. The RMS value is calculated for each frame, and the maximum *RMS* value across all frames is defined as *RMS*_max_. Since the reactive peak force is inherently coupled with the fruit’s firmness, the raw maximum frame *RMS* energy is mathematically correlated with the impact force. To eliminate this effect, the recorded force sensor data was utilized to normalize the energy feature: the raw *RMS*_max_ of each percussion was divided by its corresponding peak excitation force (*F_peak_*). This force-normalized maximum frame *RMS* energy was ultimately adopted as the input variable for the machine learning model, thereby eliminating systematic errors induced by percussive amplitude variations.

(2) $$NRMS_{\max}=\frac{RMS_{\max}}{F_{peak}}$$

The vibration decay time is defined as the duration required for the vibration signal’s amplitude to attenuate from its peak value *A*_peak_ to 1/e of the peak. To extract this feature, the absolute value of the raw vibration signal is first computed to obtain its envelope. Using a sliding window of 50 samples, the time of the peak on this envelope curve (*t*_peak_) is identified. Subsequently, the algorithm locates the end time, *t*_end_, at which the post-peak amplitude remains continuously below the attenuation threshold. The vibration decay time is then simply derived from the difference between these two time points.

(3) $$t_{\mathrm{decay}}=t_{\mathrm{end}}-t_{\mathrm{peak}}$$

where *t*_peak_ represents the time of the amplitude peak, and *t*_end_ denotes the time when the amplitude decays to 1/e of the peak value.

#### 2.4.2. Frequency-Domain Features

According to the elastomechanics theory, the natural frequency of a spherical fruit is positively correlated with its overall elastic modulus. Since the elastic modulus directly reflects the flesh stiffness and indirectly indicates developmental maturity, frequency-domain features serve as crucial indicators for watermelon quality assessment. In this study, the fundamental frequencies of the acoustic signal from the opposite side (*f*_farsound_1st_) and that of the vibration signal (*f*_vib_1st_) were selected as key frequency-domain features.

The time-domain signal was converted into the frequency domain using the Fast Fourier Transform (FFT). Since the acquired data are discrete sampled signals, the transformation follows the Discrete Fourier Transform (DFT), expressed as:

(4) $$X[k]=\sum_{n=0}^{N-1}x(n)e^{-j\frac{2\pi }{N}nk},k=0,1,\ldots ,N-1$$

where *x*(*n*) is the discrete time-domain signal, *X*(*k*) is the frequency-domain output, *n* is the discrete time index, *k* is the frequency bin index, and *N* is the FFT length. In our discrete computation, the 500-sample raw frame was zero-padded to an FFT length of *N* = 512 (the nearest power of two) to optimize the algorithm’s efficiency.

**Figure 9:**
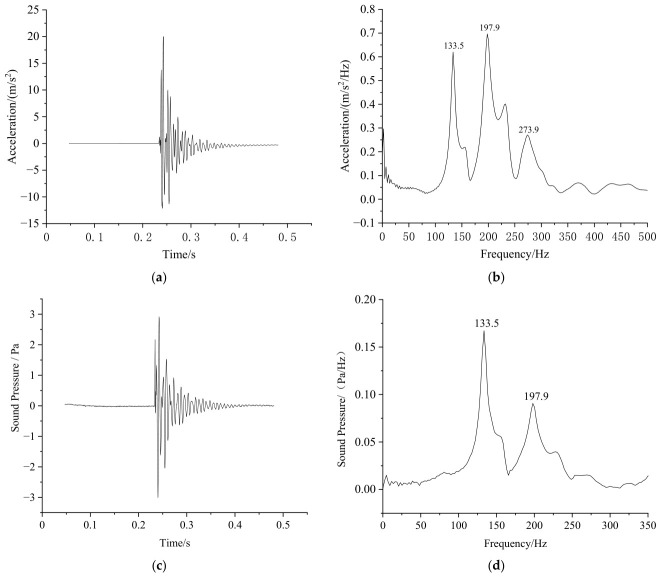
Time-domain and frequency-domain representations of vibration and acoustic signals: (**a**) time-domain waveform of vibration signal; (**b**) Fourier spectrum of vibration signal; (**c**) time-domain waveform of acoustic signal; (**d**) Fourier spectrum of acoustic signal.

The examples of acoustic and vibration signals are illustrated in Figure 9.

To comprehensively characterize the stiffness and damping properties of the fruit flesh, while mitigating collinearity between the fundamental frequency and decay time, this study constructs a composite feature parameter, denoted as *C_p_* (Crispness Proxy), calculated as follows:

(5) $$C_{p}=\frac{f_{\mathrm{vib}\_1\mathrm{st}}}{t_{\mathrm{decay}}}$$

where *f*_vib_1st_ is the fundamental frequency of the vibration signal (Hz), and *t*_decay_ is the vibration decay time (s).

This metric integrates dual information on the elastic modulus (reflecting stiffness) and damping characteristics (reflecting energy dissipation), thereby enabling a more comprehensive characterization of watermelon texture attributes. The fundamental frequency (*f*_vib_1st_) is inherently size-dependent (larger watermelons exhibit lower frequencies). However, vibration decay time (*t*_decay_) follows an inverse relationship with frequency and the internal damping ratio (ζ). By calculating their product, the geometric size dependencies mathematically cancel out, isolating the internal damping properties. Consequently, *C_p_* acts as a size-independent proxy that strictly reflects the structural integrity and cell wall turgor of the tissue, directly correlating with the sensory perception of “crispness”.

Additionally, Mel-Frequency Cepstral Coefficients (MFCC) were employed to extract nonlinear frequency envelopes that align with human auditory perception. MFCC can precisely capture subtle timbral differences in impact sounds among watermelons of varying qualities, making them highly sensitive to microstructural variations in the flesh tissue. During MFCC extraction, a standard 13-dimensional MFCC feature matrix was generated. To avoid overfitting caused by introducing high-dimensional features into a relatively small dataset, a linear correlation analysis was performed between the variance and mean value of each MFCC dimension and the CQI. The variance of the second coefficient (MFCC_2_var_) was selected as the final input feature because it exhibited the highest correlation with the quality grades, while the remaining dimensions were excluded.

### 2.5. Data Analysis Methods

#### 2.5.1. Factor Analysis of Mixed Data for Constructing the Comprehensive Quality Index (CQI)

Factor Analysis of Mixed Data (FAMD) is a multivariate statistical technique specifically designed to explore data with both quantitative and qualitative variables. Because watermelon quality is a complex, multidimensional concept, relying solely on single indicators such as SSC or firmness is insufficient to comprehensively capture the actual consumer sensory experience. Moreover, objective physicochemical metrics and subjective sensory evaluations often exhibit a certain degree of multicollinearity. To streamline the predictive targets of the model without forcing inappropriate linear assumptions on categorical data, this study employed FAMD to reduce the dimensionality of four key indicators: ripeness stage, SSC, firmness, and overall sensory evaluation score. This process established a robust and statistically rigorous benchmark for comprehensive quality assessment.

Unlike traditional methods that require numerical encoding for qualitative attributes, FAMD inherently handles mixed data types. In our evaluation matrix, SSC, firmness, and sensory scores were treated as quantitative continuous variables, whereas ripeness was strictly retained as a qualitative categorical variable. During the computation, the continuous variables were internally standardized, while the categorical ripeness variable was encoded into a disjunctive data table. Subsequently, the first principal dimension (denoted as Dim1), which accounts for the maximum proportion of total variance across all mixed-type features, was extracted to globally characterize the comprehensive quality of the watermelons.

To enhance the interpretability of the evaluation outcomes, the calculated Dim1 scores were normalized to a 0–100 scale using Min-Max scaling, thereby constructing the Comprehensive Quality Index (CQI). The expression is given as:

(6) $$CQI=\frac{Dim1-Dim1_{\min}}{Dim1_{\max}-Dim1_{\min}}\times 100$$

where *Dim*1_min_ and *Dim*1_max_ represent the minimum and maximum values of the original *Dim*1 scores, respectively.

Finally, the watermelon samples were classified into quality grades. Based on the distribution characteristics of CQI values and practical grading requirements, the samples were categorized into three quality levels to serve as classification targets for subsequent machine learning models: *CQI* ≥ 75 denotes High-Quality; 40 ≤ *CQI* < 75 denotes Medium-Quality; and *CQI* < 40 denotes Low-Quality.

#### 2.5.2. Extra-Trees Prediction Model and Parameter Configuration

In non-destructive watermelon quality assessment, acoustic and vibration signals acquired through excitation inevitably contain a certain amount of background noise and are influenced by individual sample variations. Owing to the high cost of destructive testing, the total sample size is relatively limited. Considering these constraints of small datasets coupled with feature noise, this study employs the Extremely Randomized Trees (Extra-Trees) algorithm to construct a comprehensive quality classification model.

The Extra-Trees algorithm is an ensemble learning method based on decision trees. Its core principle is to aggregate predictions from multiple decision trees to enhance model generalization. Unlike Random Forest, Extra-Trees utilizes the entire training set for constructing each individual tree, thereby maximizing information extraction from limited data. During node splitting, the algorithm generates split thresholds completely at random for each feature. This approach effectively reduces model variance and improves noise robustness, making it particularly suitable for modeling acoustic and vibration signals contaminated by environmental and equipment noise.

For model construction, the six features extracted in Section 2.4 serve as input variables: *NRMS*_max_, *t*_decay_, *f*_farsound_1st_, *C_P_*, *MFCC*_2_*var*_ and watermelon mass (*m*). The output targets comprise three comprehensive quality grades defined in Section 2.5.1: High-Quality, Medium-Quality, and Low-Quality.

Given the relatively high dimensionality of acoustic and vibration features and limited sample size, moderate pre-pruning was applied to prevent overfitting. The specific hyperparameters were configured as follows: the number of base estimators (n_estimators) set to 100, the maximum tree depth (max_depth) limited to 8, the minimum samples per leaf (min_samples_leaf) set to 3, and the maximum features (max_features) using the square-root rule. Additionally, due to class imbalance among quality grades, the class weight balancing mechanism (class_weight = ‘balanced’) was enabled. This adaptively adjusts penalty weights for minority classes, ensuring an unbiased decision boundary.

To identify the optimal model for the experimental conditions, three other machine learning classifiers were developed for performance comparison: Support Vector Classifier (SVC), Random Forest (RF), and Light Gradient Boosting Machine (LightGBM). All models utilized the exact same input features, output targets, and data partitioning scheme as the Extra-Trees model.

#### 2.5.3. Physically Constrained Data Augmentation, Cross-Validation, and Evaluation Metrics

To objectively evaluate the generalization performance of the model, this study employed stratified 3-fold cross-validation. This method maintains the proportion of High-Quality, Medium-Quality, and Low-Quality watermelons in each fold consistent with the overall sample distribution when partitioning training and testing sets, thereby avoiding evaluation bias caused by class imbalance.

Given that Medium-Quality watermelons constitute the minority class in the sample distribution (19 samples, accounting for 21%), direct training may lead to insufficient recognition capability for this minority class. To address this issue, data augmentation under physical constraints was applied to Medium-Quality watermelons within the internal loop of cross-validation to expand the minority sample size. In each iteration, two folds (approximately 58 samples) served as the original training set, within which Medium-Quality watermelon samples were augmented by a factor of 2 for model training, while one fold (approximately 29 samples) was reserved for testing. The mean of the three test results was taken as the final performance metric of the model. To prevent data leakage during training, all augmentation operations were performed exclusively on the training set of the current fold, with no modifications applied to the testing set, ensuring the objectivity of the evaluation results.

The data augmentation strategy on the extracted feature space comprises three steps, each designed in accordance with the physical characteristics of watermelon acoustic and vibration signals:

Step 1: Simulating background noise. For frequency-domain features, random noise following a uniform distribution U(−5, 5) Hz is added to the original feature values to simulate the inevitable electrical signal fluctuations and environmental interference during sensor acquisition. A non-negativity constraint is applied to ensure physical plausibility.

Step 2: Global feature scaling. All feature values of the original sample were multiplied by *λ*_1_, where *λ*_1_ follows a uniform distribution *U*(0.9, 1.1). This achieves proportional scaling of the feature matrix mathematically while also simulating the overall fluctuations in acoustic and vibration signals caused by variations in individual watermelon size and internal characteristics.

Step 3: Local stretching of time-domain features. Given that impact signals exhibit temporal broadening characteristics, two time-domain features—vibration decay time and force-normalized maximum frame *RMS* energy—were multiplied by *λ*_2_, where *λ*_2_ follows a uniform distribution *U*(0.95, 1.05). This operation enriches sample diversity in the temporal dimension while preserving the physical correlations among features.

Through this targeted augmentation strategy, the number of original Medium-Quality watermelon samples in each training fold could be doubled, achieving approximate balance with other categories in the training set.

For comprehensive model performance evaluation, average accuracy under cross-validation was employed for overall assessment, while precision, recall, macro-averaged F1 score (Macro-F1), and confusion matrix were utilized to quantitatively analyze the specific recognition performance for each quality grade.

## 3. Results

### 3.1. Analysis of the Watermelon Comprehensive Quality Index (CQI) Based on FAMD

**Figure 10:**
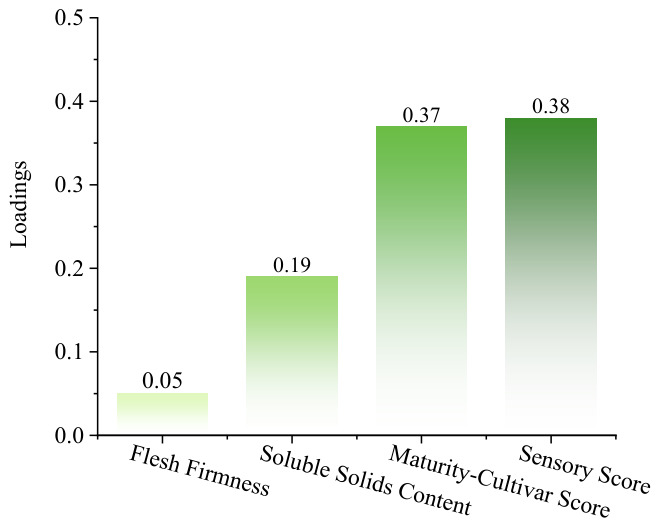
The loading values of each quality factor contribute to the overall quality.

Factor Analysis of Mixed Data (FAMD) was performed on the ripeness scores, SSC, firmness, and sensory evaluation scores of 87 watermelon samples. The extracted first principal dimension (Dim1) yielded a variance contribution rate of 30.49%, indicating that it captures nearly 30% of the variance from the original indicators. To investigate the specific impact of each individual indicator on the comprehensive quality, the eigenvector loadings of Dim1 were extracted, with the weight analysis presented in Figure 10.

As illustrated in Figure 10, the loading values for all four indicators are positive, indicating a positive correlation with the comprehensive quality. Specifically, the overall sensory evaluation and ripeness scores exhibit the highest weights (approximately 0.38 each). This prominence arises because the ripeness status and subjective tasting experience provide a reflection of the watermelon quality. They are followed by SSC (approximately 0.19), while firmness carries the lowest weight (approximately 0.05). The relatively lower weights of SSC and firmness can be attributed to their nature as localized quality factors, which are highly susceptible to variations in internal sampling locations and individual growth differences. Consequently, substituting a single indicator with a dimensionality-reduced comprehensive index can more comprehensively and stably reflect the true watermelon quality.

**Figure 11:**
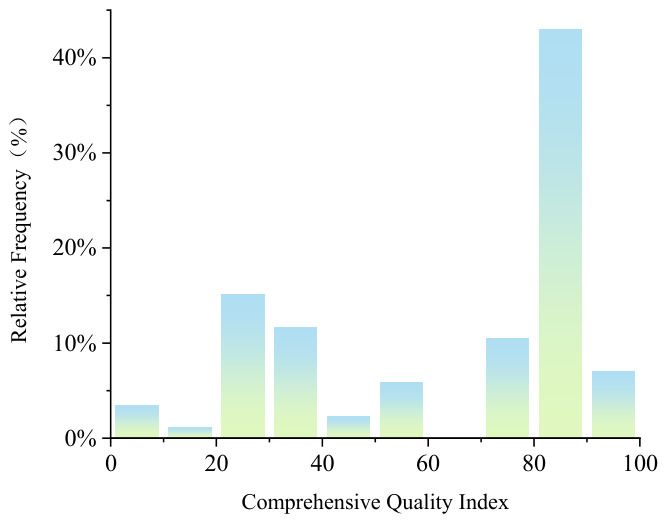
CQI frequency distribution histogram.

To facilitate the establishment of classification targets and intuitively display the results, the Dim1 scores were normalized to a 0–100 scale via Min-Max scaling to obtain the Comprehensive Quality Index (CQI). The frequency distribution histogram and the key statistics of the CQI for all samples are presented in Figure 11 and Table 1, respectively.

The comprehensive quality scores of the samples exhibit an overall bimodal distribution, with a large number of samples concentrated in the 20–40 and 70–100 point ranges, and fewer samples in the intermediate range. Based on the trough positions in the data histogram, thresholds of 40 and 75 points were established to define the quality grades. In the final grading results, there were 52 High-Quality samples (CQI ≥ 75), accounting for 59%; 8 Medium-Quality samples (40 ≤ CQI < 75), accounting for 9%; and 27 Low-Quality samples (CQI < 40), accounting for 31%. The sample distribution among the three categories was slightly imbalanced. The relative sparsity of the Medium-Quality samples provided the rationale for applying data augmentation methods in the subsequent model training.

### 3.2. Analysis of Extra-Trees Prediction Results

**Figure 12:**
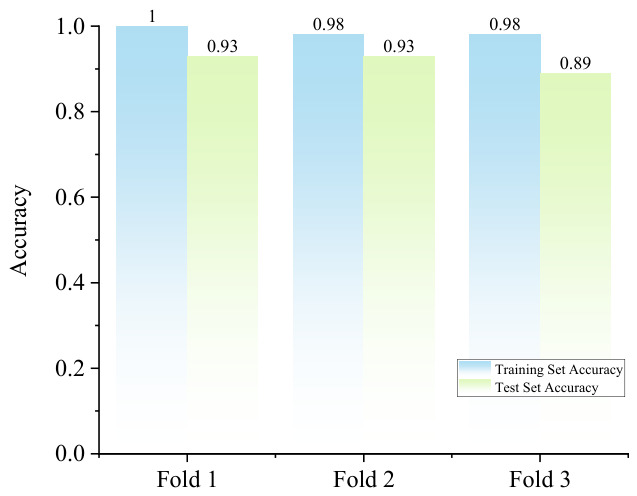
Comparison of accuracy rates between the training set and test set in 3-fold cross-validation.

Following the clear definition of quality grading labels, stratified 3-fold cross-validation was employed to evaluate model performance. The overall results are illustrated in Figure 12. The average accuracy of the model on the training and testing sets was 0.99 and 0.92, respectively, demonstrating the strong feature learning and generalization capabilities of the Extra-Trees model.

The overall classification metrics are listed in Table 2. It can be observed that the model achieved an overall accuracy of 0.92, with a Macro-F1 score of 0.80 and a Weighted-F1 score of 0.91, reflecting good overall performance. Notably, the recall for High-Quality and Low-Quality watermelons reached 1.00 and 0.93, respectively. This suggests that the model is highly precise in classifying samples at the two extremes and that acoustic and vibration signals offer high recognizability for both. This result is consistent with the biological rule that ripe and overripe watermelons exhibit significant differences in flesh cell wall strength and fiber structure [35].

**Figure 13:**
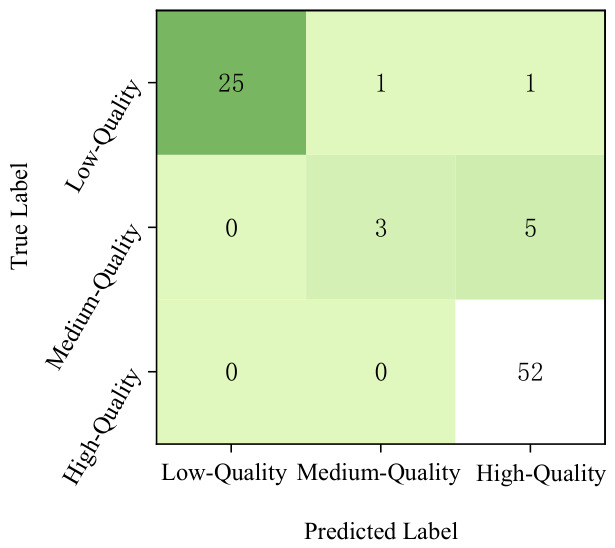
Overall confusion matrix of the test set.

However, even after balancing the data distribution through data augmentation, the model’s ability to recognize Medium-Quality watermelons remained weak, with a recall of only 0.38. As seen in the confusion matrix for the testing set (Figure 13), out of eight actual Medium-Quality samples, five were misclassified as High-Quality, and only three were correctly identified. This is because the development of agricultural product quality is typically a continuous and gradual process. As an intermediate transition state, the physical boundary between the tissue structures of Medium-Quality and High-Quality watermelons is blurred. From the perspective of acoustic and vibration features, although the natural frequencies of Medium-Quality (165–200 Hz) and High-Quality watermelons (130–150 Hz) are predominantly concentrated in distinct bands, a small number of transitional samples fall into the intermediate 150–165 Hz range, resulting in a slight overlap in their overall distributions. and their maximum frame root-mean-square energy (*RMS*_max_) distribution ranges are also closely adjacent, causing an overlap in the acoustic feature space [27]. Consequently, even with increased training samples via data augmentation, it remains difficult for the model to fully distinguish between these two classes of samples with similar quality.

In addition to classification performance, the computational efficiency of the proposed Extra-Trees model was also assessed. Although the current system remains in the algorithmic proof-of-concept stage, the time for a single detection cycle—encompassing feature extraction and model inference—was evaluated on a standard computer. The average processing time per sample is approximately 1.4 s, demonstrating the algorithm’s high computational efficiency and its strong feasibility for future real-time online sorting applications.

### 3.3. Predictive Performance Comparison of Different Machine Learning Models

In addition, a Support Vector Classifier (SVC), Random Forest (RF), and Light Gradient Boosting Machine (LightGBM) were employed for comparison with the proposed Extra-Trees model. The detailed results are presented in Table 3.

As shown in Table 3, all evaluated models achieved exceptionally high classification accuracies on the testing set, ranging from 0.88 to 0.92. This uniform high performance strongly indicates that the extracted acoustic-vibration features are highly separable and robust. Although the accuracy differences among the models became relatively marginal under the current dataset, the Extra-Trees model still achieved the highest testing accuracy of 0.92.

### 3.4. Analysis and Validation of Acoustic Signals

**Figure 14:**
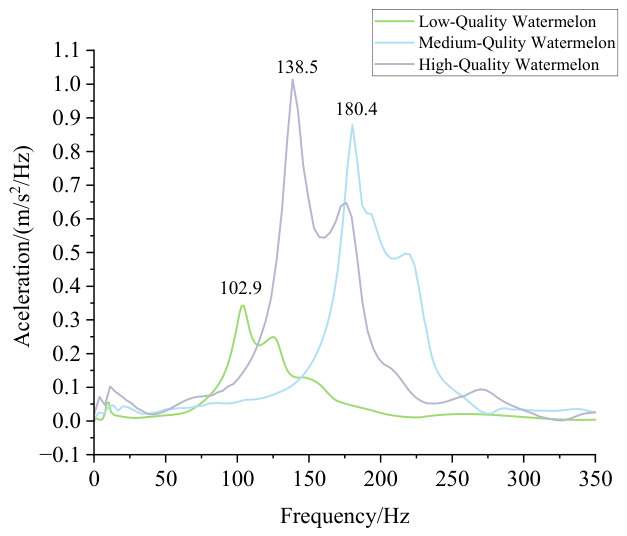
Comparison of typical frequency-domain signals of watermelons with different quality grades.

The accuracy of the model fundamentally stems from the distinct acoustic and vibration characteristics exhibited by watermelons of different qualities. According to the elastomechanics theory, the natural frequency of a spherical fruit is positively correlated with its elastic modulus. This relationship can be expressed as $f\propto \sqrt{E/m}$ (where *f* denotes the natural frequency, *E* represents the elastic modulus, and *m* is the mass). The firmness of the flesh directly influences its stiffness (*K*) and elastic modulus (*E*); a firmer texture corresponds to a higher elastic modulus and, consequently, a higher natural frequency. As observed in the frequency-domain signal comparison of watermelons across different quality grades (Figure 14), distinct gradient differences exist in their fundamental frequencies. For High-Quality watermelons, the internal tissues are fully developed with uniform cell structures. Their signals exhibit a fundamental frequency peak with a pronounced amplitude around 130–150 Hz, and the acoustic response generated by impact excitation is relatively crisp and resonant.

The fundamental frequency peak of Medium-Quality watermelons shifts slightly to the right, concentrating primarily in the 165–200 Hz band. This shift occurs because the Medium-Quality category includes some unripe fruits and lower-quality ripe fruits; fruit firmness during the early developmental stages is slightly higher than in later stages, thereby producing a higher-frequency acoustic response [36]. Conversely, the signals of Low-Quality watermelons form a smaller fundamental frequency peak around 80–100 Hz, which is situated far from the distribution ranges of the other two grades, resulting in a distinctly muffled acoustic response upon impact. This dullness is attributed to severe over-ripening or even spoilage in Low-Quality watermelons; the extensive degradation of pectin and cellulose leads to cell separation and significant flesh softening [35].

**Figure 15:**
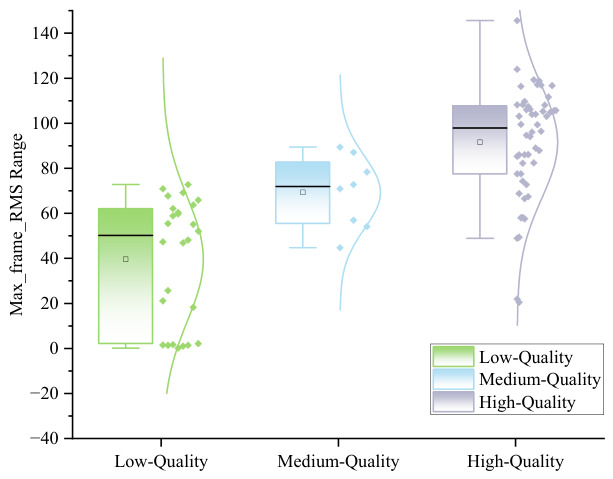
Distribution of the force-normalized maximum frame *RMS* energy (*NRMS*_max_) for watermelons of different quality grades.

Regarding time-domain features, the distribution of the force-normalized maximum frame *RMS* energy (*NRMS*_max_) is illustrated in Figure 15. As the comprehensive quality of the watermelon increases, the *NRMS*_max_ values exhibit a step-wise upward trend. Because High-Quality watermelons possess intact internal cell development and continuous fiber structures, energy dissipation during acoustic wave transmission is minimal, yielding generally higher *NRMS*_max_ values. In contrast, Low-Quality watermelons exhibit internal sponginess or cracks that form a strong acoustic-damping medium. This causes a sharp attenuation in signal amplitude, concentrating their *NRMS*_max_ values in the lowest range. The *NRMS*_max_ values for Medium-Quality watermelons fall between the two extremes, overlapping somewhat with the High-Quality distribution. This trend corroborates the analytical results of the fundamental frequency, jointly reflecting how the internal structural integrity of the watermelon influences the propagation characteristics of acoustic and vibration signals.

## 4. Discussion

This study constructed a non-destructive prediction model for comprehensive watermelon quality based on acoustic and vibration signals and machine learning methods. The experimental results demonstrate that the Extra-Trees model exhibits satisfactory overall predictive capabilities, with extremely high recall rates for High-Quality and Low-Quality watermelons, thereby verifying that acoustic and vibration signals can achieve effective quality grading. However, the decision ambiguity between Medium-Quality and High-Quality watermelons, as exposed by the confusion matrix, also reveals the limitations of acoustic and vibration signals in the fine-grained detection of watermelon quality.

The model performed excellently in identifying Low-Quality and High-Quality watermelons. This occurs because distinct physical changes take place within the internal structure during the ripening or deterioration process. However, the recall rate for Medium-Quality watermelons was only 0.38, with five out of eight Medium-Quality samples misclassified as High-Quality. This phenomenon can be attributed to the following two factors:

First, the physical differences between these grades are minimal. With the development of modern agricultural breeding technologies in China, the baseline quality of commercial watermelons in the market has significantly improved. The High-Quality and Medium-Quality watermelons defined in this study exhibit minimal differences in cell wall structure and flesh density. Their quality distinction primarily depends on the accumulation of soluble solids content (SSC). The propagation characteristics of acoustic waves are mainly influenced by the density and elastic modulus of the medium. Studies have shown that during the young fruit stage, sugars produced by photosynthesis are mainly used for building cell walls, respiratory consumption, and the metabolic synthesis of other substances. The ripening and softening of the fruit are caused by the degradation of pectin and cellulose mediated by cell wall-related enzymes. Thus, the dynamic changes in SSC and firmness represent a highly complex biochemical process [36]. Consequently, it is difficult to distinguish such complex and subtle compositional changes solely relying on acoustic and vibration signals.

Second, individual biological variations introduce interference. As biological agricultural products, individual variations in watermelons—such as rind thickness, internal cavity size, and fruit shape proportions—produce non-linear interference with acoustic waves and vibrations. This inevitably leads to random fluctuations in the acoustic and vibration responses of Medium-Quality and High-Quality watermelons. From the perspective of frequency-domain distribution, although there is a range difference between the fundamental frequency peaks of Medium-Quality (165–200 Hz) and High-Quality (130–150 Hz) watermelons, they overlap at the boundaries. The distribution of *RMS*_max_ values in the time domain also exhibits partial overlapping. This overlap in the feature space makes it difficult for the model to fully distinguish between these two classes of similar-quality samples, even when employing data augmentation strategies.

Compared with existing research, the innovations of this study are reflected in the following aspects:

(1) Construction of a more comprehensive evaluation system. Previous studies often used only SSC or firmness as a single ripeness label [2], ignoring the actual sensory experience of consumers. By combining ripeness, SSC, firmness, and sensory evaluation through Factor Analysis of Mixed Data (FAMD), this study constructed the Comprehensive Quality Index (CQI), making the evaluation results align more closely with actual consumer demands.

(2) Adoption of a physically meaningful data augmentation strategy. To address the issue of a small sample size, this study performed data augmentation by simulating environmental background noise, globally scaling feature values, and locally stretching time-domain features. This approach effectively expands the minority class samples while preserving the physical meaning of the original signals, providing a practical method for sample expansion in the non-destructive testing of agricultural products.

(3) Further validation of model applicability on acoustic and vibration datasets. By comparing four models (Extra-Trees, RF, LightGBM, and SVC), it was found that our data preprocessing strategy provides a robust foundation for quality assessment. However, the Extra-Trees algorithm is still considered the most suitable choice for this study. Its extreme randomized splitting mechanism and full-sample utilization intrinsically provide stronger resistance against overfitting on small datasets containing physical noise. It also requires less hyperparameter tuning, making it highly reliable for real-time sorting.

In practical fruit wholesale or retail scenarios, the most critical need for both merchants and consumers is to accurately eliminate Low-Quality watermelons to avoid economic losses and poor experiences. The requirement for distinguishing between High-Quality and Medium-Quality watermelons is relatively relaxed. The model in this study achieved a recall rate of 0.96 for Low-Quality watermelons, enabling the highly effective screening of spoiled fruits. Although a portion of Medium-Quality watermelons was classified as High-Quality, this is generally considered an acceptable and reasonable error in actual sales. Therefore, this classification model and its results meet the practical demands of fruit grading and sales, demonstrating clear value for real-world application.

## 5. Conclusions

This study used “Beijing Tianwang” and “Shanxi Xiabao” watermelons as experimental subjects to investigate a non-destructive testing method for comprehensive watermelon quality. The research aimed to address the limitations of relying on a single physicochemical indicator for comprehensive quality evaluation, as well as the overfitting problem common to small-sample acoustic and vibration signals in complex classification tasks. Through feature extraction, data augmentation, and multi-model comparisons, the main conclusions are as follows:

(1) Established a comprehensive watermelon evaluation system that aligns with consumer demands. To overcome the limitations of previously relying solely on SSC or firmness as a single grading criterion, this study employed Factor Analysis of Mixed Data (FAMD) to integrate ripeness scores, SSC, firmness, and subjective tasting scores through dimensionality reduction. This process generated the Comprehensive Quality Index (CQI), which objectively categorized the samples into three grades: High-Quality, Medium-Quality, and Low-Quality. This index comprehensively reflects multiple factors influencing watermelon quality, providing reliable labels for machine learning classification.

(2) Validated the physical correlation between acoustic and vibration features and watermelon quality. Watermelons of different quality grades exhibited significant gradient differences in their fundamental frequency and force-normalized maximum frame *RMS* energy (*NRMS*_max_). For High-Quality watermelons, the fundamental frequency is concentrated around 130–150 Hz, accompanied by the highest *NRMS*_max_ values. For Low-Quality watermelons, the fundamental frequency decreased to 80–100 Hz, presenting the lowest *NRMS*_max_ values. The values for Medium-Quality watermelons fell between these two extremes. These patterns are consistent with elastomechanics theory, providing a physical basis for non-destructive acoustic and vibration testing.

(3) Developed a quality classification prediction model based on the Extremely Randomized Trees (Extra-Trees) algorithm. Under small-sample experimental conditions, and combined with physically constrained data augmentation strategies such as simulating background noise and scaling feature values, the Extra-Trees model achieved an overall accuracy of 0.92 on the testing set. The recall rate was 0.93 for Low-Quality watermelons and 1.00 for High-Quality watermelons, demonstrating effective watermelon quality grading.

(4) Verified the applicability of the Extra-Trees model on small-sample acoustic-vibration datasets through multi-model comparisons. Parallel comparisons with Random Forest (RF), Light Gradient Boosting Machine (LightGBM), and Support Vector Classifier (SVC) demonstrated that all evaluated baseline models achieved excellent predictive performance. Among them, the Extra-Trees model achieved the optimal performance (accuracy of 0.92) under the current experimental conditions. Due to its extreme randomized splitting mechanism, it effectively suppressed overfitting and provided the most robust and computationally efficient classification for this small-sample dataset.

Limited by the variety and quantity of samples, the model’s generalization capability still requires cross-variety validation in future work. Furthermore, the classification ambiguity between Medium-Quality and High-Quality watermelons, caused by their similar physical characteristics, reflects a physical bottleneck: acoustic waves are insensitive to microscopic chemical changes. Finally, the random errors introduced by manual impact excitation and sensor attachment methods need to be mitigated through the deployment of automated devices.

## Figures and Tables

**Table 1:** Descriptive statistics of CQI.

CQI Statistics	Score
Mean	63.49
Std	28.79
Min	0.00
25th percentile	32.76
50th percentile	80.07
75th percentile	86.71
Max	100.0

**Table 2:** Classification metrics of the Extra-Trees model on the test set.

Test Set Classification Metric	Precision	Recall	F1-Score	Support
Low-Quality Watermelon	1.00	0.93	0.96	27
Medium-Quality Watermelon	0.75	0.38	0.50	8
High-Quality Watermelon	0.90	1.00	0.95	52
Accuracy	-	-	0.92	87
Macro avg	0.88	0.77	0.80	87
Weighted avg	0.92	0.92	0.91	87

**Table 3:** Comparison of classification performance among different machine learning models.

Model	Training Set Accuracy Rate	Test Set Accuracy Rate
Extra-Trees	0.99	0.92
SVC	0.97	0.88
LightGBM	0.99	0.91
RandomForest	0.97	0.92

## Data Availability

The data presented in this study are available upon request.
